# Fe/MOF based platform for NIR laser induced efficient PDT/PTT of cancer

**DOI:** 10.3389/fbioe.2023.1156079

**Published:** 2023-03-30

**Authors:** Zixing Liang, Xiaofeng Li, Xiaofang Chen, Jiawei Zhou, Yanan Li, Jianhui Peng, Zhousheng Lin, Gai Liu, Xiancheng Zeng, Cheng Li, Lifeng Hang, Hailiang Li

**Affiliations:** ^1^ Guangdong Second Provincial General Hospital, Guangzhou, China; ^2^ Guangzhou First People’s Hospital, School of Medicine, South China University of Technology, Guangzhou, China; ^3^ Jinan University, Guangzhou, China; ^4^ Hainan General Hospital, Hainan Affiliated Hospital of Hainan Medical University, Hainan, China

**Keywords:** tumor, microenvironment, MOF, photodynamic therapy, photothermal therapy, nanoenzyme

## Abstract

**Introduction:** Photodynamic therapy (PDT) and photothermal therapy (PTT) are widely used in the treatment of tumors. However, their application in the treatment of clinical tumors is limited by the complexity and irreversible hypoxia environment generated by tumor tissues. To overcome this limitation, a nanoparticle composed of indocyanine green (ICG) and Fe-MOF-5 was developed.

**Methods:** We prepared F-I@FM5 and measured its morphology, particle size, and stability. Its enzyme like ability and optical effect was verified. Then we used MTT, staining and flow cytometry to evaluated the anti-tumor effect on EMT-6 cells *in vitro*. Finally, the anti-tumor effect *in vivo* has been studied on EMT-6 tumor bearing mice.

**Results:** For the composite nanoparticle, we confirmed that Fe-MOF-5 has the best nanozyme activity. In addition, it has excellent photothermal conversion efficiency and generates reactive oxygen species (ROS) under near-infrared light irradiation (808 nm). The composite nanoparticle showed good tumor inhibition effect *in vitro* and *in vivo*, which was superior to the free ICG or Fe-MOF-5 alone. Besides, there was no obvious cytotoxicity in major organs within the effective therapeutic concentration.

**Discussion:** Fe-MOF-5 has the function of simulating catalase, which can promote the decomposition of excessive H_2_O_2_ in the tumor microenvironment and produce oxygen to improve the hypoxic environment. The improvement of tumor hypoxia can enhance the efficacy of PDT and PTT. This research not only provides an efficient and stable anti-tumor nano platform, but also has broad application prospects in the field of tumor therapy, and provides a new idea for the application of MOF as an important carrier material in the field of photodynamic therapy.

## Graphical Abstract

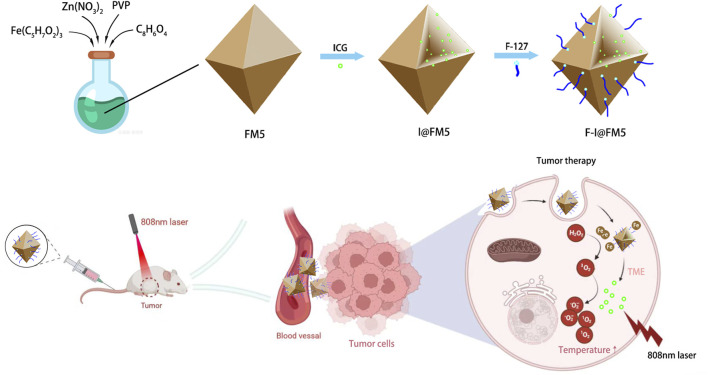

## 1 Introduction

Photodynamic therapy (PDT) and photothermal therapy (PTT) would prove to be the effective modality for tumor therapy (Li et al., 2018a; Goel et al., 2018; Zhang et al., 2018; Zhao et al., 2018), which have attracted considerable attention owing to its accurate target positioning and low systemic toxicity. In PDT, photosensitizer, illumination, and oxygen are indispensable (Wong et al., 2005). At first, after the photosensitizers gather around the diseased tissue, we will irradiate the diseased tissue with an appropriate light source. The photosensitizer absorbs energy and then transitions from the ground state to the excited state. The excited state photosensitizer transfers energy to the oxygen molecules around the cell, and then a series of photochemical reactions occur, producing a large number of highly oxidizing reactive oxygen species (ROS) (Pogue et al., 2003). The oxidation of these ROS can damage biomolecules and structures in tumor cells, thereby killing tumor cells and achieving the effect of tumor treatment. PTT is a treatment method that gathers materials with high photothermal conversion efficiency near tumor tissue and converts light energy into heat energy under the irradiation of external light to kill cancer cells (Jaque et al., 2014; Chen et al., 2019). However, the O_2_ content of tumor tissue largely determines the therapeutic efficacy of PDT and PTT (Donohoe et al., 2019; Yang et al., 2021). It is well known that hypoxia is a major feature of solid tumors (Barker et al., 2015; Courtnay et al., 2015; Meng et al., 2018), that is because the malignant proliferation of tumor cells is always faster than the formation of vascular network. When the vascular network in the tissue cannot meet the needs of tumor cell growth and metabolism, a local hypoxic microenvironment is formed. Clearly, increasing O2 concentration of tumor tissues may improve the phototherapy efficacy (Cheng et al., 2015; Tang et al., 2016; Gao et al., 2018).

In recent years, with the continuous development of nanotechnology, nanomaterials have been widely used in tumor treatment. Because of their special physical and chemical properties, nanomaterials can modify various functional groups or targeting groups, promote biocompatibility and carry multiple heavy components (Rosenblum et al., 2018; Xie et al., 2019a; Ali et al., 2021). In addition, the small size of nanomaterials coupled with their enhanced penetration and retention effect (EPR) (Li et al., 2018b; Luo et al., 2021), enable them to reach and enrich tumor tissues conveniently. As a member of nanomaterials, the design and synthesis of metal-organic frameworks (MOFs) have become the most attractive research direction in recent decades due to their special structure and potential applications in many fields (Liu et al., 2021). MOFs are kind of porous crystalline material, which are the network structure crystal formed by composition of metal ions or clusters with organic ligands. They have regulatable porous structure and highly ordered structure. Due to the intrinsic properties of MOFs, they are widely used in catalysis (Huang et al., 2017), drug delivery (Ding et al., 2022), optics, and sensing (Olorunyomi et al., 2021).

As we know, high concentration of hydrogen peroxide (H_2_O_2_) is one of the most notably features of the tumor microenvironment (TME) (Feng et al., 2018; Castaldo et al., 2019; Lin et al., 2019), which is obviously different from healthy tissues. Considering that the decomposition of H_2_O_2_ may improve the hypoxia of TME, so as to improve the efficiency of PDT and PTT. Therefore, the use of nanoenzyme-coated photosensitizers to promote the therapeutic effect may be a promising strategy. Zhang prepared a series of well-defined MOF (MOF-5, FeII-MOF-5, FeIII-MOF-5) hollow nanocages by a facile solvothermal method (Zhang et al., 2014), without any additional supporting template. Fe-MOF has great stability and a hollow structure, which can contain photosensitizers (Zhao et al., 2020), drugs (Guo et al., 2021) or other components (Li et al., 2020; Shi et al., 2021). In addition, Fe-MOF has peroxidase-like activity due to the presence of Fe (Brozek and Dincă, 2013).

Herein, an F127-ICG@Fe-MOF-5 NPs (F-I@FM5), which possesses nanozyme activity and PDT/PTT ability, was applied to tumor combination therapy with significantly strength of mutual enhancement. We also evaluated the efficacy of the combination therapy *in vivo* and *in vitro*. Consistent with our assumptions, F-I@FM5 shows the characteristics of high curative effect on tumor and low toxicity, providing a safe and effective solution for the field of tumor treatment.

## 2 Results and discussion

### 2.1 Characterization of nanoparticles

The preparation method of Fe-MOF-5 (FM5) referred to the previous literature (Zhang et al., 2014), and then we use FM5 as the main scaffold of nanoparticles. After fully reacting with ICG and F-127, we get I@FM5 and F-I@FM5. To evaluate the success of the synthesis of nanoparticles and their characterization, we measured the morphology, particle size, absorbance, and other aspects.

**FIGURE 1:**
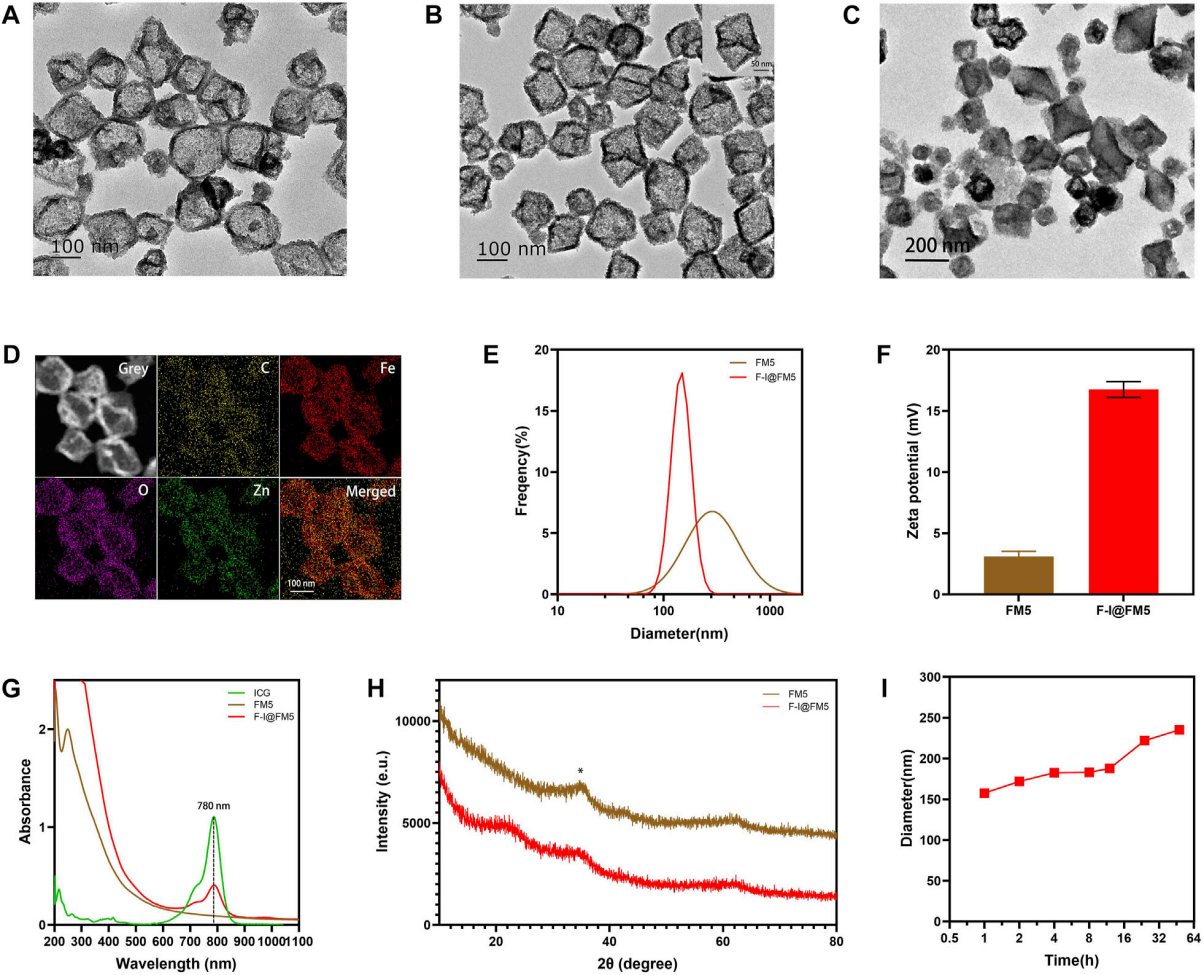
Preparation and characterization of F-I@FM5. **(A)** TEM images of I@FM5 (Scale bar equal to 100 nm). **(B)** TEM images of F-I@FM5 (scale bar 100 nm, scale bar in the inset 50 nm). **(C)** TEM images of F-I@FM5 treated with an acid solution (scale bar 200 nm). **(D)** EDS mapping of Fe, Zn, O and C, respectively. **(E)** Dynamic light scattering analysis of FM5 (478 nm) and F-I@FM5 (156.52 nm). **(F)** Zeta potentials of FM5 and F-I@FM5. **(G)** Absorption spectra of ICG, FM5, and F-I@FM5. Characterized peaks of ICG at 780 nm were observed in F-I@FM5, implying the successful construction of F-I@FM5. **(H)** Size stability of F-I@FM5 in PBS. **(I)** XRD patterns of FM5 and F-I@FM5, respectively.

First, we observed the morphology of nanoparticles by transmission electron microscope (TEM). Figure 1A shows the TEM image of I@FM5, which had an irregular polyhedron structure, and their sizes were about 150–170 nm. It is consistent with the morphology of common Fe-based MOFs (Wang et al., 2022a; Yao et al., 2022). After modified F-127, the morphology of F-I@FM5 was also tested in the same way. Compared with that before modification, the nanoparticles did not show obvious deformation and damage (Figure 1B). Previous studies have shown that most MOFs have the characteristics of accelerated degradation in the acidic environment (Ma et al., 2022), which will be beneficial to the release of the drug contained, so that we placed F-I@FM5 in the solution of pH 6.5 for 24 h, some frameworks were destroyed under TEM (Figure 1C), which meant that ICG could be better released in the acidic environment of TEM. To further qualitatively analyze the elements of nanoparticles, the energy-dispersive X-ray spectroscope (EDS) mapping was determined, and the results presented in Figure 1D. We found the good distribution of C, Fe, O, Zn elements in the structure of nanoparticles, which is consistent with the molecular composition of the raw materials we used. Meanwhile, these elements are evenly distributed, which means that the materials fully react in the synthesis process of F-I@FM5.

The hydrodynamic diameter of I@FM5 was 478 nm tested by the dynamic light scattering (DLS) technique (Figure 1E), while the F-I@FM5 obtained after F-127 modification was 156.52 nm. It is not consistent with the particle size shown in the TEM. That is because FM5 are easy to gather in an aqueous solution to form precipitation due to hydrophobic interaction, so the average particle size detected is the diameter of several nanoparticles after aggregation. As a surface active substance, F-127 can increase the hydrophilicity of nanoparticles and help them to disperse uniformly in solution (Xie et al., 2019b). When F-127 is used to modify the I@FM5, the diameter the of single nanoparticle can be measured. According to previous research, nanomaterials with size of 60–400 nm show better permeability and retention (EPR) effect in tumor tissue (Fan et al., 2021; Du et al., 2022). It means that the nanoparticles we designed can be retained and accumulated in tumor tissue. Moreover, The small size demonstrated that the prepared particles were suitable for systemic drug administration route (Sun et al., 2022). In addition, compared with FM5, the zeta potential of F-I@FM5 is higher (Figure 1F), which shows that after F-127 modification, our nano-system becomes more difficult to aggregate, which is consistent with the previous reason for the smaller hydrodynamic diameter of F-I@FM5. Next, we measured the absorption spectra of ICG, FM5 and F-I@FM5. The characteristic peak of ICG at 780 nm was also determined in F-I@FM5, further proving that ICG has been successfully encapsulated in FM5 (Figure 1G). We then measured the concentration of ICG in the supernate obtained by the reaction solution, and calculated that the loading efficiency (LE) of ICG was 32.8% and encapsulation efficiency (EE) of ICG was 49%. We also studied the release behavior of ICG in neutral and acidic solutions, the results showed that F-I@FM5 was able to release more ICG in the acidic environment (Supplementary Figure S1), which indicated that when the nanoparticles reached the tumor, ICG could be released in the tumor to induce PDT and PTT treatment in the acidic environment around the tumor. X-ray powder diffraction (XRD) patterns were used to characterize the crystal structure of FM5 and F-I@FM5. We found that the XRD peaks of FM5 recorded at 2θ = 36.34^°^ could be well consistent with the literature we refer to, and after the decoration of F-127, the peaks of F-I@FM5 matches well with FM5. It indicated the successful synthesis of F-I@FM5 (Figure 1H).

As we know, stability of nanoparticles is a crucial requirement for the treatment in the body (Ding et al., 2015). The hydrodynamic size changes of F-I@FM5 in phosphate buffered saline (PBS) were measured in 48 h to demonstrate its stability. As shown in Figure 1I, no significant change of size could be observed, suggesting that F-I@FM5 possessed a suitable stability profile for further investigation.

### 2.2 Nanozyme activity assay

**FIGURE 2:**
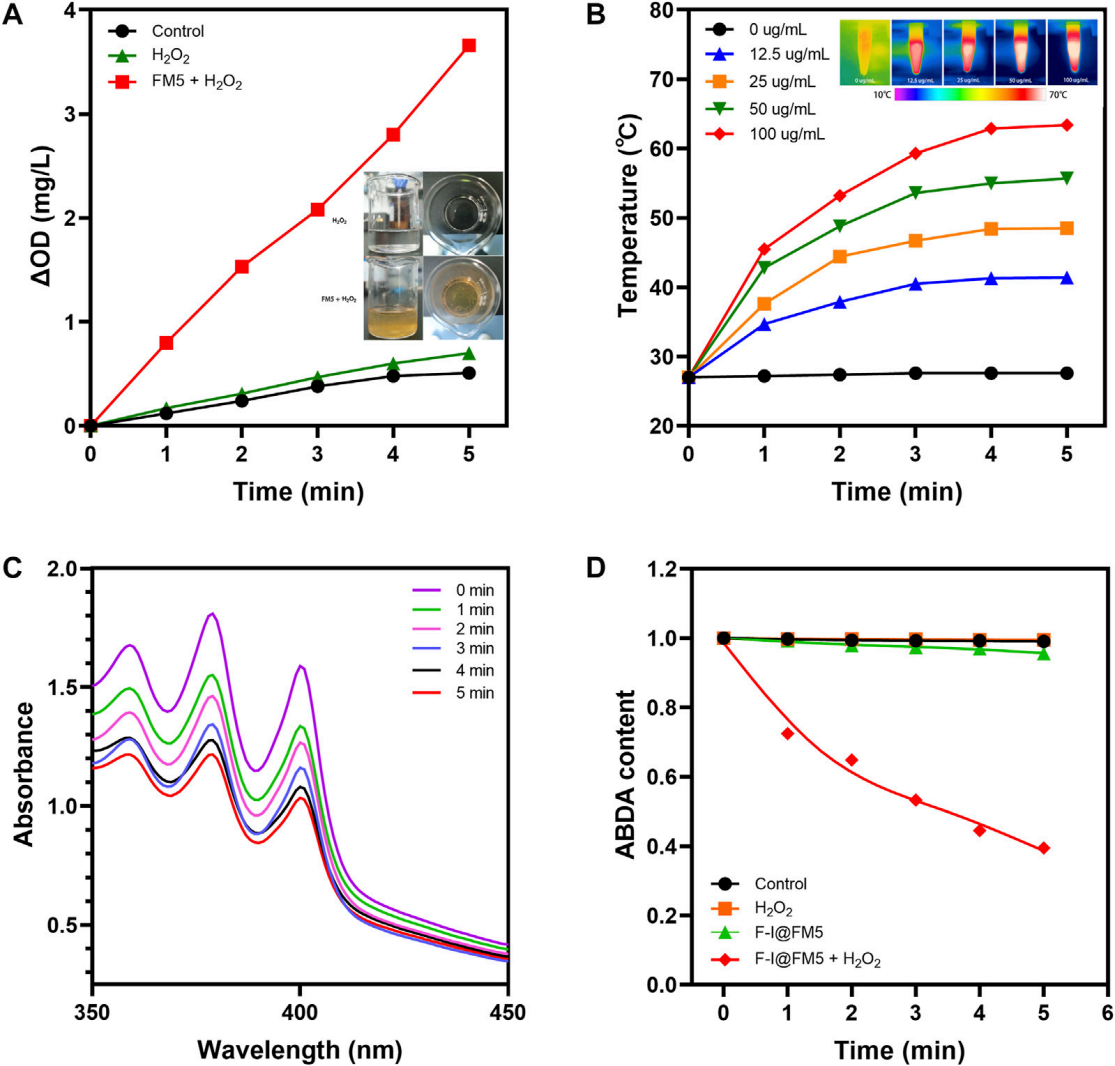
Physical and chemical properties F-I@FM5. **(A)** Time-dependent changes of dissolved O_2_ concentrations in different groups. Insert map is Bubble generation in solutions. **(B)** Temperature changes of F-I@FM5 with different concentrations under NIR. Inset map is the infrared images of different groups after 5 min of illumination. **(C)** Absorption spectra of ABDA at different times in F-I@FM5 + H_2_O_2_ group. **(D)** Changes of ABDA content in different groups.

As reported in previous studies, the hypoxic tumor microenvironment is one of the important reasons for the unsatisfactory efficacy of PDT in the treatment of solid tumors, because oxygen is the main factor in the process of PDT (Wu et al., 2021a; Zhang et al., 2022). As we know, there is a large amount of H_2_O_2_ accumulated in the TME (Wu et al., 2021b). In order to improve hypoxia, the catalytic ability to decompose H_2_O_2_ is indispensable. Xi Xiang had designed a Fe-MOF based bio-/enzyme-mimics nanoparticle for the treatment of cancer, which showed great catalytic ability due to the existence of Fe (Xiang et al., 2021). There was a large amount of Fe in components of FM5, so we thought it might have similar enzyme activity. The catalytic ability of FM5 was determined by adding FM5 and H_2_O_2_ to oxygen-free water and measuring the content change of oxygen. As shown in Figure 2A, compared with H_2_O_2_ or FM5 alone, the oxygen content in the H_2_O_2_+FM5 group was significantly increased. Meanwhile, a large number of oxygen bubbles were observed in the H_2_O_2_+FM5 group. The results showed that because of the existence of Fe in FM5, it had the same enzyme-like ability as other MOF-based nanozymes (Wang et al., 2020a), which could promote the decomposition of H_2_O_2_ to improve the hypoxic environment of tumors.

### 2.3 *In vitro* photothermal property

We know that ICG can strongly absorb light energy to convert it into heat energy (Li et al., 2019). In order to evaluate whether ICG can maintain the characteristics of laser-induced temperature rise after being loaded into FM5, we conducted photothermal conversion experiments in different groups. In the previous absorbance spectrum, ICG showed good absorbance near 780 nm wavelength, so we chose 808 nm near-infrared light (NIR) for irradiation. F-I@FM5 at different concentrations (0, 12.5, 25, 50, 100 μg/mL of ICG) were irradiated with 808 nm laser (1 W/cm^2^). The temperature of F-I@FM5 dispersion (100 μg/mL) rise from 27°C to 63.4°C after being irradiated with 808 nm laser for 5 min (Figure 2B). In contrast, for the FM5 and PBS, no apparent temperature change could be observed at the same condition, indicating the F-I@FM5 with good photothermal conversion property. In terms of tumor therapy, to the best of our knowledge, PTT exerts anti-tumor effects mainly through direct thermal ablation (over 42°C) (Hou et al., 2018). After 5 min of irradiation, F-I@FM5 (50 μg/mL) could rise from 27°C to 55.7°C, which represented that the temperature change achieved by F-I@FM5 was enough to kill tumor cells.

### 2.4 *In vitro* ROS generation

PDT is a treatment mode for local treatment of diseases based on the interaction of light, photosensitizer and oxygen (Correia et al., 2021). The production of reactive oxygen species (ROS) plays a major role in PDT and the production of ^1^O_2_ is closely related to the oxygen concentration (Liu et al., 2019). According to the previous nanozyme activity experiments, FM5 can promote the decomposition of H_2_O_2_ to produce oxygen. In addition, ICG has been proven to be an excellent material for PDT (Yang et al., 2022). Therefore, we can infer that under 808 nm laser, F-I@FM5 can show good active ROS generation ability. In order to evaluate the effect of F-I@FM5 on ROS generation, 9,10-Anthracenediyl-bis (methylene) di malonic Acid (ABDA), a probe with irreversibly reduced absorption in the presence of singlet oxygen (He et al., 2020), was used to detect the generation of ROS. As shown in Figure 2C, under 808 nm laser after 5 min, the control group and H_2_O_2_ group did not influence on ABDA degradation, and the absorbance of ABDA had a little decline in F-I@FM5 group. After adding H_2_O_2_ in F-I@FM5, the absorption of ABDA in solution displayed a continuous decrease to 39% of the initial absorbance upon laser irradiation, indicating that the O_2_ generated from the H_2_O_2_ decomposition could increase the ^1^O_2_ generation (Figure 2D). It demonstrated that F-I@FM5 was not inferior to other methods to increase the ROS, such as the transportation of oxygen (Wang et al., 2017) or catalase (Xu et al., 2020) and photosensitizer to tumor sites. This is because F-I@FM5 can improve tumor hypoxia by converting H_2_O_2_ enriched in the TME into O_2_, thus promoting ^1^O_2_ generation and improving the photodynamic therapy effect. Therefore, F-I@FM5 had the potential to kill tumor cells.

### 2.5 Cell uptake experiment

**FIGURE 3:**
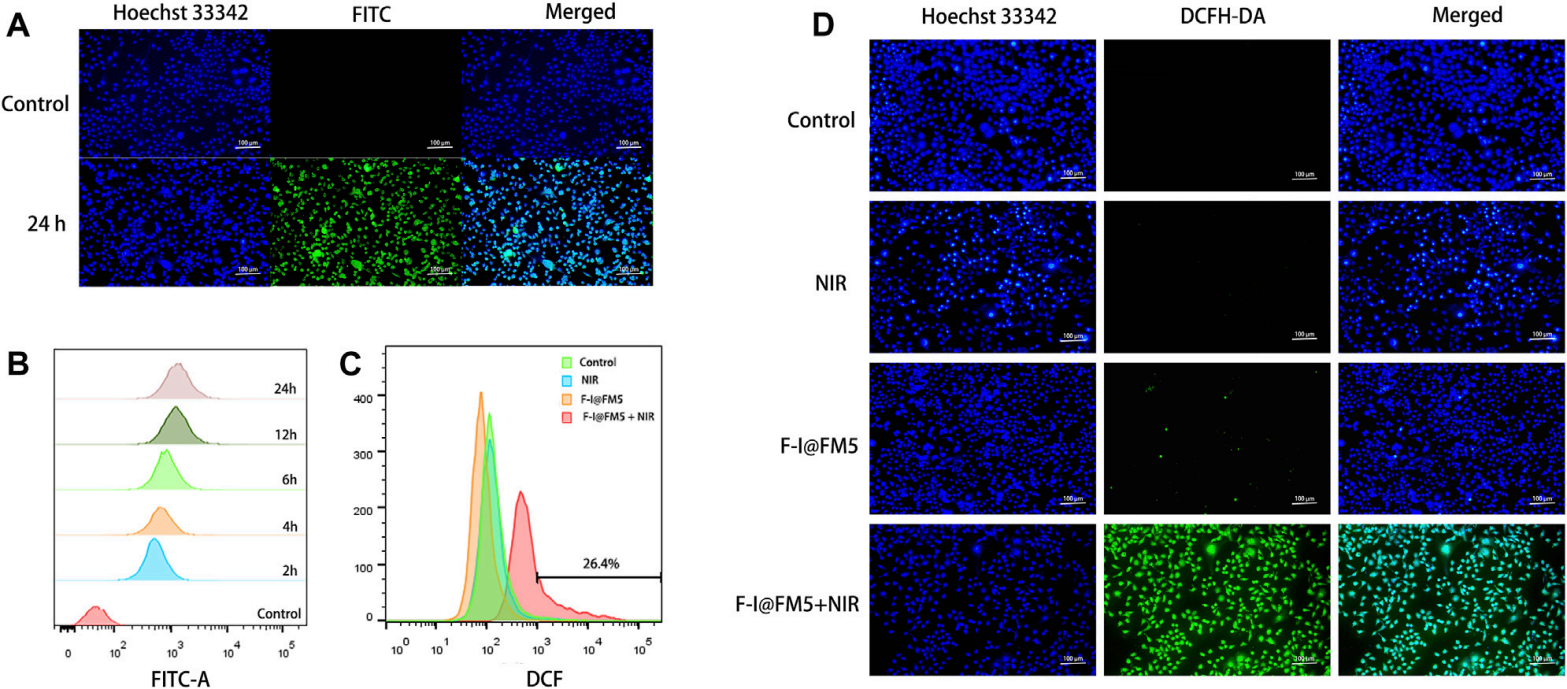
Cell uptake and ROS generation of F-I@FM5 *in vitro*. **(A)** Observation of intracellular FITC after the nanoparticles and EMT-6 cells were incubated for 24 h. **(B)** Flow data of intracellular FITC fluorescence at different periods. **(C)** Flow data of intracellular DCF fluorescence. **(D)** Observation of intracellular DCF in different groups (Scale bar equal to 100 nm).

Before conducting cell experiments, we studied the uptake behavior of cells to nanoparticles. Because ICG is a near-infrared fluorescent dye, the Olympus inverted fluorescence microscope and flow cytometer we used cannot detect it. We used fluorescein isothiocyanate isomer (FITC) instead of ICG to load into FM5, and F-F@FM5 was obtained after modification with F-127. When the nanoparticles enter the cell, the cell will emit FITC-specific green fluorescence. The distribution and intensity of FITC fluorescence can be analyzed by flow cytometry and fluorescence inversion microscope to reflect the uptake of nanoparticles by the cell. At FITC concentration of 50 μg/mL, the nuclei were stained with Hoechst33342 after the nanoparticles and EMT-6 cells were incubated for 24 h. It can be seen from Figure 3A that all cells emit strong green fluorescence, which indicates that the nanomaterials have been endocytosed by the cells. In addition, the results of flow cytometry were consistent with those of fluorescence staining. As shown in Figure 3B, we had used fluorescence intensity of FITC to mediately quantify the uptake of the particles. We could find that after 6 h of co-incubation, the uptake of F-I@FM5 was significantly higher than the control groups. With the increase of incubation time, FITC fluorescence become strongly. Compared with 6 h, it was observed that the cell uptake increased nearly twofold when after 24 h incubation. These findings confirmed F-I@FM5 can successfully pass through the cell membrane and accumulate in cells.

### 2.6 Intracellular ROS generation

As we know, the generation of active oxygen is very dependent on the presence of oxygen (Cheung and Vousden, 2022). In the previous study, Wang designed a Co@Fe_3_O_4_ nanozymes, which can induce apoptosis of human renal tumor cells (A-498) by catalyzing the decomposition of H_2_O_2_ to generate a ROS burst (Wang et al., 2019). Based on the photothermal efficiency and enzyme activity of F-I@FM5, we expect that it can also improve the hypoxia of TME, thus generating large number of ROS. To evaluate the ROS generation in EMT-6 cells, Dye 2,7-dichlorouoresce diacetate (DCFH-DA) was used as an indicator to detect the ROS production ability of living cells. This is because DCFH-DA can be converted into DCFH in living cells, and DCFH can be oxidized by ROS to 2,7-Dichlorofluorescein (DCF), which produces obvious green fluorescence in cells (Jin et al., 2019). First, we observed the fluorescence production of DCF in EMT-6 cells by flow cytometry. As shown in Figure 3C, compared with other groups, the count of cells with DCF fluorescence in the F-I@FM5+NIR group increased significantly in flow cytometry. Consistently, In addition, the image obtained by fluorescence microscope is consistent with the result of flow cytometry (Figure 3D), the green fluorescence intensity of the F-I@FM5+NIR group incubated under light was stronger than that of another group, which proved that the F-I@FM5+NIR group could produce more ROS, which was consistent with the results of *in vitro* ROS detection and cell uptake experiments. It can be seen from the above results that treatment with F-I@FM5 leads to the ROS burst, which was expected to enhance ability of anti-tumor of F-I@FM5.

### 2.7 *In vitro* anti-tumor effect

**FIGURE 4:**
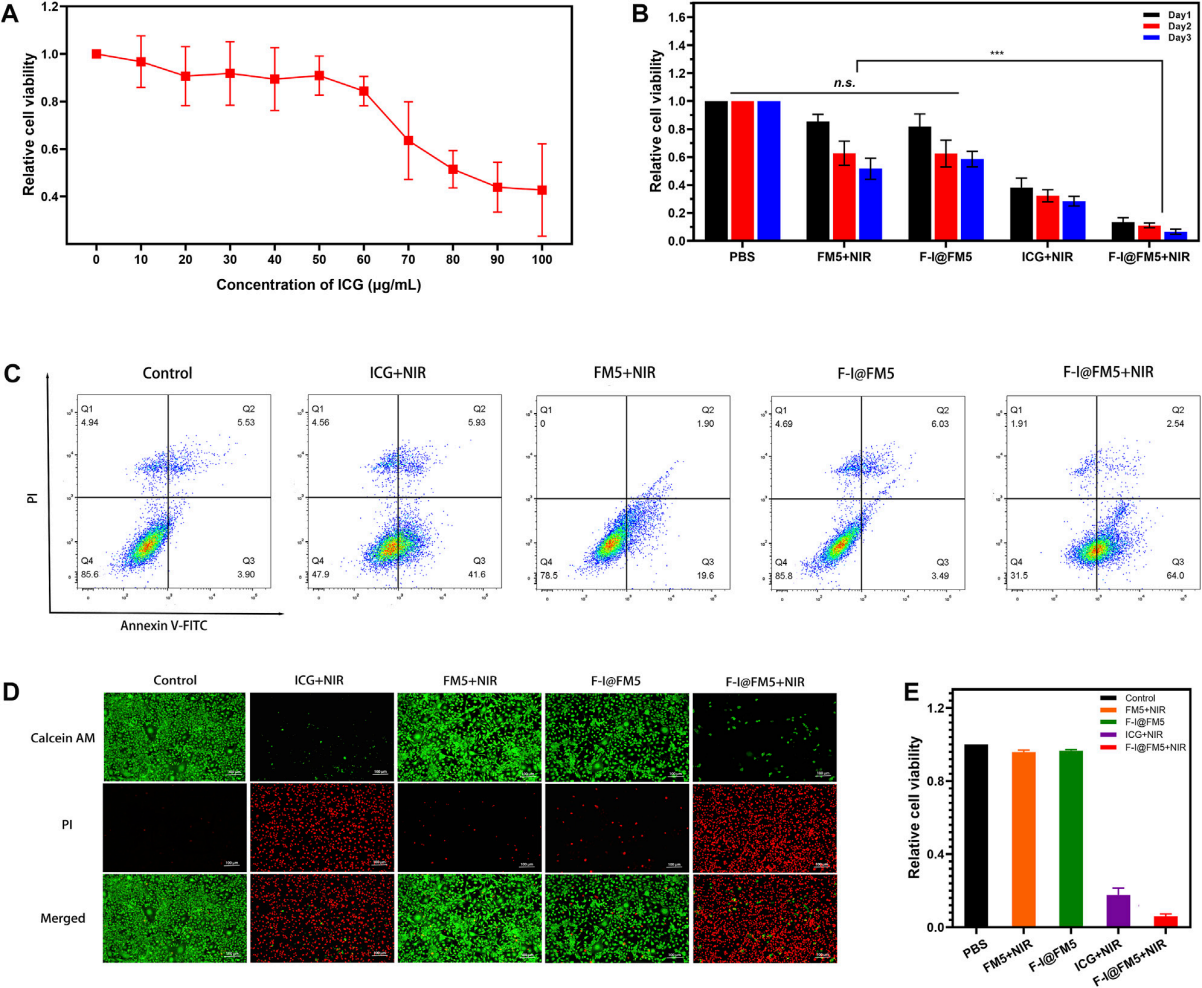
Anti-tumor activities of F-I@FM5 *in vitro*. **(A)** Relative cell viability with increasing ICG concentration of F-I@FM5. **(B)** Anti-tumor activities of F-I@FM5 *in vitro*. Relative cell viability in different groups (*** *p* < 0.001, *n* = 5). **(C)** Flow cytometry data of EMT-6 cells co-stained with Annexin V-FITC/PI of various treatments. **(D)** Images of EMT-6 cells co-stained with Calcein-AM and PI of various treatments (Scale bar equal to 100 nm). **(E)** Relative cell viability obtained by the fluorescent pictures.

In order to determine the cytotoxicity of F-I@FM5, MTT method was used to measure the effect of F-I@FM5 at different concentrations on tumor cells without NIR. As shown in Figure 4A, it can be observed that when the ICG concentration less than 70 μg/mL, F-I@FM5 have no obvious effect on cell activity. However, when the concentration of ICG exceeds 70%, the cell vitality decreases significantly. In order to avoid the damage to normal cells caused by F-I@FM5, we need to select a concentration less than 70 μg/mL for follow-up study.

Subsequently, the tumor suppressive ability of F-I@FM5 was evaluated under laser irradiation conditions (808 nm, 1 W/cm^2^, 10 min). As shown in Figure 4B, the relative cell viability of ICG + NIR group was significantly lower than that of PBS, FM5+NIR and F-I@FM5 groups, which showed that when ICG reached a certain concentration, PDT/PTT triggered by ICG could cause certain damage of tumor cells. It was worth noting that compared with the ICG + NIR group, the relative cell viability of the FM5+ICG group decreased more significantly. This result confirmed the efficacy of the nanoparticles, F-I@FM5 could improve the hypoxia of the tumor microenvironment and further promote the effect of PDT/PTT. At the same time, it showed that the effect of the ICG would not be lost after being encapsulated in the hollow mesopore of FM5.

Then we analyzed the therapeutic effect of F-I@FM5 at the cell level by flow cytometry and AM/PI cell staining. Annexin V-FITC/PI staining flow cytometry was used to further detect apoptosis. As shown in Figure 4C, apoptotic cells and necrotic cells of F-I@FM5 +In NIR group accounted for 68.4%, while Control, FM5+NIR and F-I@FM5 group were 14.4, 21.5% and 14.2%, respectively. It was represents that under 808 nm laser irradiation, F-I@FM5 can destroyed majority of tumor cells. In order to obtain intuitive information of living and dead cells, Calcein AM/PI staining experiments were performed on EMT-6 cells. Living cells and dead cells emit green and red fluorescence, respectively. As shown in Figure 4D, an intuitive image of cells could be observed using a fluorescent inverted microscope. It was worth noting that compared with other groups, more red fluorescence was detected in the F-I@FM5+NIR treatment group, indicating that the number of dead cells increased. The relative cell viability obtained by counting the dead and alive cells in the fluorescent pictures was consistent with the MTT assay (Figure 4E). This phenomenon indicated that under the nanozyme activity of F-I@FM5, the efficiency of PDT and PTT against tumor cells was enhanced, which was consistent with the results obtained by MTT method. This fluorescence staining experiment further verified the synergistic therapeutic effect of F-I@FM5.

In conclusion, these results reflect that the physical and chemical properties of F-I@FM5 are well reflected in tumor cells. It enhances the tumor suppressive ability of PDT and PTT, which has certain advantages in cancer treatment compared with PDT or PTT alone. This is consistent with the current research direction and consensus (Wu and Yang, 2017). Through multifunctional nano carriers, the stability of MOF can enhance the accumulation of drugs at the tumor site and slow down its metabolism. Meanwhile, it can also produce synergistic effects with drugs or photosensitizers through the multiple properties of MOF and its derivatives, so as to better treat tumors.

### 2.8 *In vivo* anti-tumor effect

**FIGURE 5:**
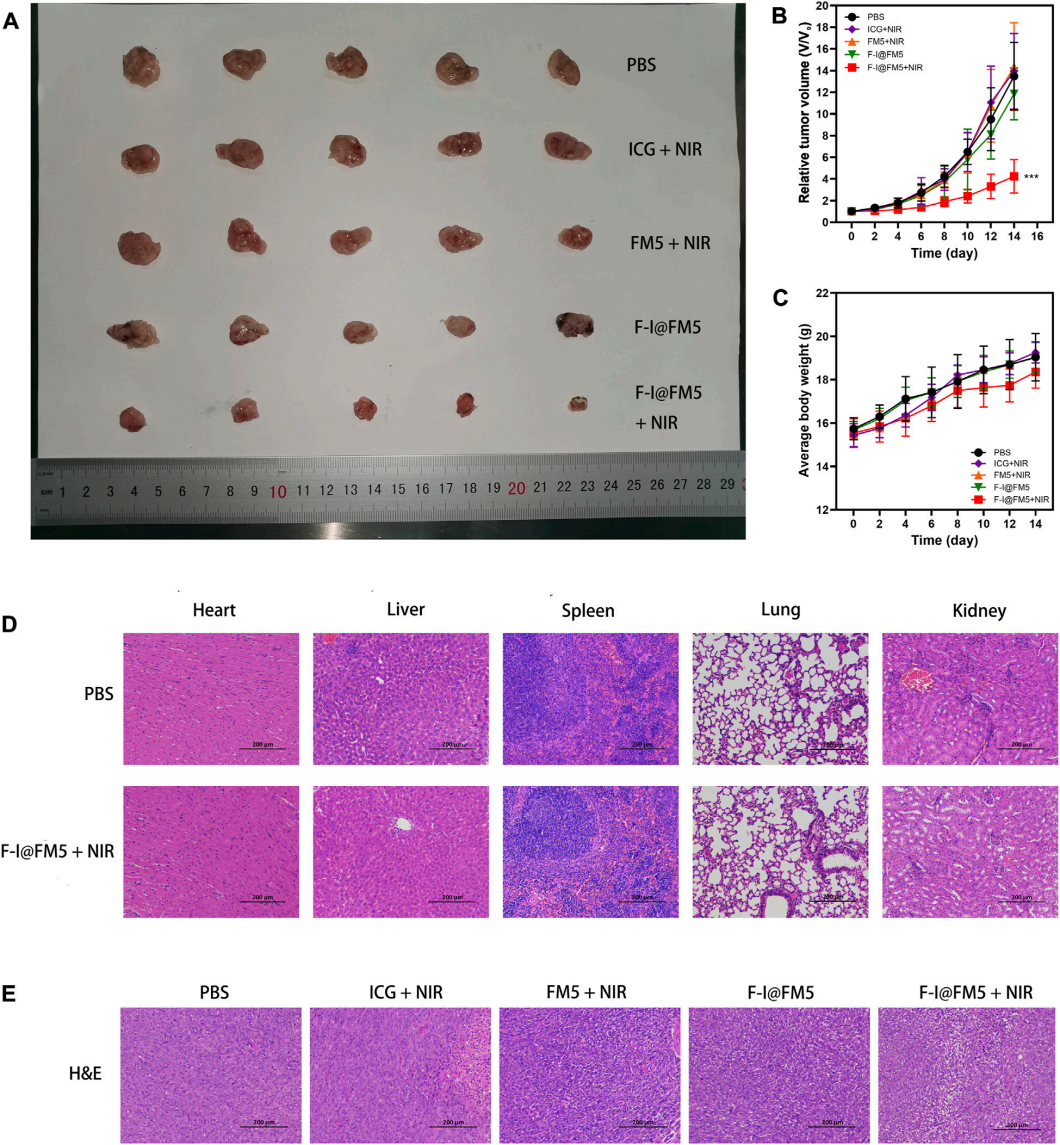
Anti-tumor activities of F-I@FM5 in EMT-6 tumor-bearing mice. **(A)** Photograph of harvested tumor from mice after 14 d of different treatments. **(B)** Tumor growth curves of mice during the 14 days treatment. **(C)** Animal weight changes of different groups during the treatment period. Error bars represent mean ± SD (*p < 0.05, **p < 0.01, ***p < 0.001. *n* = 5 per group). **(D)** Histological observation of major organs for safety determination by comparing the F-I@FM5+NIR group to the normal group. **(E)** Histological observation of the tumor tissues with H&E staining from different treatment groups of mice.

As shown in Figures 5A, B, fast tumor growth was observed in the control untreated group. We found that the tumor growth rate in the ICG + NIR groups was similar to that in the control group, which was different from the tumor inhibition effect *in vitro*. This is due to the fast metabolic efficiency of free ICG in the body. Generally, after being injected into the body, ICG would be cleared by the liver within 150–180 s (Sucher et al., 2020), and there were few drugs finally reaching the tumor, so it had no obvious inhibition effect on tumor cells. The tumor rate of F-I@FM5 without NIR was slightly smaller than that of the control group, but there was no statistical difference, which indicated that within the effective concentration of treatment, F-I@FM5 had no obvious toxicity to cells, which was consistent with the MTT assay. Compared with other groups, we observed the strongest anti-tumor effect with F-I@FM5+NIR group, which can prove that due to the synergistic effect of the ICG mediated PDT/PTT and the hypoxia improved by FM5, the growth rate of the tumors will decrease. The advantages of F-I@FM5+NIR therapy further confirmed the benefits of transporting therapeutic drugs through nano carriers (Zheng et al., 2021). Until the end of treatment, the average body weight of all groups did not reveal any noticeable trend of weight loss (Figure 5C). Moreover, the H&E staining of tissue sections of major organs showed no obvious tissue damage when compared to the untreated group (Figure 5D), demonstrating that the nanoplatform had no cytotoxic effect on other normal tissues *in vivo*. H&E staining of tumor sections showed that compared with other groups, the density of tumor cells in the F-I@FM5+NIR group was decreased (Figure 5E). Therefore, the designed F-I@FM5 had great potential in PDT/PTT synergistic therapy, which was consistent with our expectations.

However, compared with other nano multi-function platforms (Jiang et al., 2018; Wang et al., 2020b), the inhibition of tumor in the tumor model was not as obvious as that of cell experiments. After further analysis, we thought the reason was that the F-I@FM5 lacks tumor-specific targeting. It is common knowledge that the EPR effect can increase the accumulation of local drug concentration. Compared with active targeting (Wang et al., 2022b), the delivery efficiency of EPR is lower, which is not enough to make the drug completely distributed in the tumor tissue. Moreover, due to the specificity of the tumor microenvironment, the tumor has a certain resistance to temperature changes and ROS, leading to the failure to eliminate all tumor cells.

In the further research, some targeted groups should be attached to the surface of nanomaterials, which enable nanoparticles to reach tumors through active targeting, thereby improving the therapeutic efficiency of tumors. In addition, PDT/PTT combined with anti-tumor drugs was also a promising direction. At the same time, with the increase of nano components, there would inevitably be more metabolic disorders and toxic. It was necessary to strengthen the monitoring of biological distribution in the process of tumor treatment, so as to provide more effective strategies for tumor treatment and avoid toxicity to other tissues. At present, the research on nano materials to improve the tumor microenvironment is still in the initial stage. With the continuous deepening of research, it is believed that the multi-functional nano therapy system can achieve great success in tumor therapy.

## 3 Conclusion

In summary, this study successfully developed F-I@FM5, as a new nano conformal material, promoted the production of local oxygen in tumors and improved the therapeutic efficiency of tumors by cooperating with PDT and PTT. The simulated catalase activity of FM5 was utilized to react with the endogenous hydrogen peroxide of TME to generate oxygen to combat hypoxia. In addition, the ICG was used to convert O_2_ into ROS to kill tumor cells. This new nanocomposite could effectively improve tumor hypoxia, improve the efficiency of PDT and PTT, and showed a better photodynamic effect. The nanoparticles proposed in this paper provide an effective strategy for enhancing anticancer therapy.

## 4 Material and methods

### 4.1 Material

Iron acetylacetonate (Fe(C_5_H_7_O_2_)_3_), zinc nitrate (Zn(NO_3_)_2_), p-phthalic acid (C_8_H_6_O_4_), polyvinyl pyrrolidone (PVP), ethanol (C_2_H_6_O) and N, N-Dimethylformamide (DMF) were obtained from Shanghai Aladdin Biochemical Technology Co., Ltd. 9,10-Anthracenediyl-bis (methylene) di malonic Acid (ABDA), Indocyanine green (ICG) was purchased from Shanghai Macklin Biochemical Co., Ltd. Phosphate-buffered saline (PBS), Dulbecco’s modified eagle medium (DMEM), Fetal bovine serum (FBS), sodium pyruvate, essential amino acids, and non-essential amino acids were from ExCell Bio (China). Calcein-AM/PI Live Cell/Dead Cell Double Staining Kit was purchased from Beijing solarbio science & technology co.,ltd. All other reagents were from Beyotime Biotechnology (China) and used as received unless otherwise noted.

### 4.2 Preparation of F-I@FM5

Adding iron acetylacetonate (Fe(C_5_H_7_O_2_)_3_), zinc nitrate (Zn(NO_3_)_2_), p-phthalic acid (C_8_H_6_O_4_) and polyvinyl pyrrolidone (PVP) in a certain proportion into the mixed solvent of ethanol (C_2_H_6_O) and N, N-Dimethylformamide (DMF), and heat the mixture in an oil bath (110°C, 6 h) under stirring conditions. After the solution was cooled, The FM5 was obtained *via* centrifugation. The product was washed with ethanol several times, and then vacuum dried for further use.

1 mL of ICG solution (2 mg/mL in ethanol) was mixed with 1 mL of FM5 suspension (2 mg/mL in ethanol). After ultrasonic vibration for 5 min, placing the mixture in the refrigerator at 4°C for 24 h, then we get the ICG@FM5. Then, 1 mL of F-127 solution (30 mg/mL in ethanol) was mixed with 1 mL of ICG@FM5 suspension. After ultrasonic vibration for 5 min, slowly drop the mixed solution into 5 mL pure water under stirring and reacting for 10 min. After centrifugation, F-I@FM5 was obtained. Suspending the F-I@FM5 with pure water for further use.

### 4.3 Characterization of F-I@FM5

The nanostructure and size of F-I@FM5 were observed by Transmission electron microscope (JEM-2100F). The particle size and size distribution of F-I@FM5 were measured by Winner 802 nanometer particle size meter (Jn-winner). Zeta potential measurements were performed at 25°C on a Malvern Zeta Size-Nano Z instrument. UV-vis absorbance spectra of FM5, ICG, and F-I@FM5 were observed with the UV-3200S Spectrophotometer (MAPADA). X-ray diffraction (XRD) patterns of the samples were analyzed by BRUKER D8 VENTURE X-ray single crystal diffractometer. The LE and EE of ICG in F-I@FM5 were measured as follows. Following preparation, the nanoparticles were centrifuged at 7,000 rpm for 10 min, and free ICG in the supernate was measured by Ultraviolet Spectrophotometer at 780 nm. The LE and EE were calculated as LE (%) = [(weight of loaded drug)/(total weight of nanoparticles)]×100; EE (%) = [(weight of loaded drug)/(weight of initially added drug)]×100. In the ICG release experiment, we dispersed F-I@FM5 (10 μg/mL ICG) in aqueous solutions of pH 7.0 and pH 5.0, and centrifuged the solution at different time points (1, 2, 4, 8, 16, 32, 64 h). Then determined the absorbance of the supernatant. Finally, the released ICG content and the proportion were obtained through the concentration-absorbance curve of ICG.

### 4.4 Nanozyme activity assay

After using Na₂SO₃ to prepare anaerobic water, a probe of the MP516 dissolved oxygen meter (Shanghai San-Xin Instrumentation) was inserted under the surface of anaerobic water, and tightness of the system was examined first, after which the FM5 (50 μg/mL) and H_2_O_2_ (5 mM) were added with a syringe. The changes in the dissolved oxygen level were recorded at the indicated time to confirm the nanozyme activity. In addition, FM5 and H_2_O_2_ were added to pure water to observe the generation of O_2_ bubbles.

### 4.5 *In vitro* photothermal property

In order to evaluate the photothermal property of F-I@FM5 NPs, different concentrations of NPs were added to PBS. Then irradiated the solutions with 808 nm near-infrared laser (1 W/cm^2^) for 5 min and recorded the temperature change of the solutions by T3S smart phone infrared camera (Iray) per minute.

### 4.6 *In vitro* ROS generation

In order to determine the production of ROS, we use ABDA as the detection probe, because the absorbance of ABDA will be reduced after the reaction among ABDA and ^1^O_2_. In the experiment, ABDA in ethanol (50 μg/mL) and H_2_O_2_(50 μM) was added to a solution of F-I@FM5 NPs (ICG, 50 μg/mL). Then the solution was irradiated with 808 nm laser (1W/cm^2^). Recording the absorbance of ABDA at the specified time intervals after irradiation, and indirectly comparing the generation of ROS among groups by the change of absorbance.

### 4.7 Cell uptake experiment

The image of cell uptake was detected by Fluorescent Inverted microscope. In order to load FITC into FM5, 1 mL of FITC solution (1 mg/mL in ethanol) was mixed with 1 mL of FM5 suspension (1 mg/mL in ethanol) for 24 h, then we get the FITC@FM5. Then, 1 mL of F-127 solution (30 mg/mL in ethanol) was mixed with 1 mL of FITC@FM5 suspension. After ultrasonic vibration for 5 min, slowly drop the mixed solution into 5 mL pure water under stirring and reacting for 10 min. After centrifugation, F-F@FM5 was obtained. EMT-6 cells were seeded in 24-well plate at the density of 5 × 10^4^ cells/well and incubated with the F-F@FM5 (FITC, 50 μg/mL) for 24 h, then we fixed the cells with 4% paraformaldehyde and stained with Hoechst33342 for 10 min. Finally, take out the cover glass and obtain the image of the cells with Fluorescent Inverted microscope.

The uptake of nanoparticles *in vitro* was studied by flow cytometry. EMT-6 cells were seeded in 6-well plate at the density of 2 × 10^5^ cells/well and incubated with either free ICG or the F-I@FM5 NPs (ICG, 50 μg/mL) for different times. The cells were collected, and cell uptake was determined from ICG fluorescence per cell using a BD FACS Aria Ⅲ Flow cytometer and FlowJo software for analysis.

### 4.8 Intracellular ROS generation

The production of intracellular ROS was detected using fluorescent dye 2,7-dichlorofluoresce diacetate (DCFH-DA) by Fluorescent Inverted microscope or flow cytometer. EMT-6 cells were seeded in 24-well plate and allowed to adhere for 24 h. Then ells were treated with F-I@FM5 for 24 h and loaded with DCFH-DA (10 μmol/L) in dark at 37°C for 15 min. Fluorescence images were observed with OLYMPUS CKX53 Fluorescent microscope, and ROS generation of per cell using a Flow cytometer and FlowJo software for analysis.

### 4.9 Cytotoxicity and apoptosis assay

The EMT-6 cells were seeded in a 96-well plate at a density of 5,000 cells per well, and cultured overnight at 37°C in a 5% CO_2_ incubator. The next day, cells were incubated with F-I@FM5 solutions at a series of concentrations (0–100 μg/mL ICG) for 24 h under the same condition. Cell viability was evaluated by the MTT assay kit. The optical density (OD) was measured at 490 nm and recorded by a microplate reader.

To compare the tumor inhibition effect of different groups, the EMT-6 cells were incubated with parallel concentrations of FM5, ICG and F-I@ FM5 for 24 h. Subsequently, the groups with NIR were exposed to the 808 nm laser for 10 min (1 W/cm^2^), then continue to incubate cells for 24 h. Cell viability was evaluated by the MTT assay kit. The optical density (OD) was measured at 490 nm and recorded by Thermo Scientific Multiskan Sky Microplate Reader.

Phototherapeutic effect was also investigated by Calcein AM)/PI staining. Then EMT-6 cells were seeded in a 24-well plate with a density of 5 × 10^4^ cells per well. The EMT-6 cells were incubated with parallel concentrations of FM5, ICG and F-I@ FM5 for 24 h. Subsequently, the groups with NIR were exposed to the 808 nm laser for 10 min (1 W/cm^2^). The other groups were incubated under the same conditions without irradiation. Control groups in the dark were incubated in fresh DMEM medium. After removing fresh DMEM medium, Calcein AM (4 mmol/L) and PI solutions (4 mmol/L) in PBS were added to EMT-6 cells and incubated for 20 min. Finally, images of the cells were obtained by fluorescence microscope.

Flow cytometry was used to study the tumor inhibition effect of different therapy *in vitro*. EMT-6 cells were seeded in 6-well plate at the density of 2 × 10^5^ cells/well and incubated with parallel concentrations of FM5, ICG and F-I@ FM5 for 24 h. Subsequently, the groups with NIR were exposed to the 808 nm laser for 10 min (1 W/cm^2^). The cells were harvested, and incubated with AnnexinV-FITC/PI Apoptosis Detection Kit. Then apoptosis status of per cell using a BD FACSCalibur flow cytometer and FlowJo software for analysis.

### 4.10 *In vivo* therapeutic efficacy

EMT-6 cells (2 × 10^6^ cells in 100 μL of PBS) were injected into the right armpit of BALB/C nude mice (4 weeks old, 15–16 g). When the tumor size reached 70–100 mm^3^, EMT-6 tumor-bearing mice were randomly divided into 5 groups: (a) PBS, (b) ICG + NIR, (c) FM5+NIR, (d) F-I@FM5, (e) F-I@FM5+NIR. Different solutions were injected into the tail vein of the tumor-bearing mice. After 24h, tumors were irradiated by the 808 nm laser (0.5 W/cm^2^) for 10 min.

The body weight and tumor volume of tumor-bearing mice were recorded every 2 days within 14 days after irradiation. Tumor volumes were determined using the formula: V = a × (b^2^)/2, where a is the length and b is width of each tumor in mm respectively. After 14 days, the animals were killed and the tumors and main organs were collected for analysis. The histological changes of tumor tissue and main organs were evaluated by hematoxylin and eosin (H&E) staining.

## 5 Statistical analysis

All results are expressed as the mean ± SEM or SD as indicated. Comparisons among groups were analyzed *via* independent samples with the one-factor ANOVA test using SPASS 17.0 software. All statistical data were obtained using a two-tailed student’s *t* test and homogeneity of variance tests (*p* values < 0.05 were considered significant).

## Data Availability

The original contributions presented in the study are included in the article/Supplementary Material, further inquiries can be directed to the corresponding authors.
